# Cardiovascular risk in rare diseases: a prognostic stratification model in a cohort of sarcoidosis patients

**DOI:** 10.1007/s11739-023-03314-8

**Published:** 2023-05-23

**Authors:** Luigi Rizzi, Chiara Coppola, Veronica Cocco, Carlo Sabbà, Patrizia Suppressa

**Affiliations:** Department of Internal Medicine, and Rare Diseases Centre “C. Frugoni”, University Hospital of Bari, Piazza Giulio Cesare 11, 70124 Bari, Italy

**Keywords:** Rare disease, Sarcoidosis, Atherosclerosis, Cardiovascular risk, Carotid ultrasound

## Abstract

Sarcoidosis is a rare granulomatous disease that can affect any organ; as other chronic diseases, it leads to increased risk of atherosclerosis and cardiovascular (CV) disease. The aim of our observational study was to define a prognostic stratification model of sarcoidosis patients based on the evaluation of CV risk through common carotid Doppler ultrasound and cardiovascular risk scores assessment; for this reason, a clinical phenotyping of sarcoidosis patients in four subgroups was done, based on the different organ involvement. A cohort of 53 sarcoidosis patients and a cohort of 48 healthy volunteers were enrolled. Results showed that CV risk was higher in sarcoidosis cohort than in the control group when evaluated through CV risk scores and Doppler parameters: peak-systolic velocity (PSV) and end-diastolic velocity (EDV) were significantly lower in sarcoidosis cohort (*p* = 0.045 and *p* = 0.017, respectively), whereas intima media thickness (IMT) showed higher values in sarcoidosis group than in controls (*p* = 0.016). The analysis of sarcoidosis phenotypes showed no significative differences of CV risk among them when CV risk scores were considered, while partial differences emerged by evaluating subclinical atherosclerosis. Results also highlighted a relationship between CV risk score and carotid Doppler ultrasound parameters: EDV showed an inverse correlation with Framingham score (*R* = − 0.275, *p* = 0.004), whereas IMT showed a direct one (*R* = 0.429; *p* = 0.001); furthermore, an inverse correlation between PSV and EDV and illness duration (*R* = − 0.298, *p* = 0.030 and *R* = − 0.406, *p* = 0.002, respectively) was found, so suggesting a higher CV risk in patients with a longer story of disease.

## Introduction

Sarcoidosis is a chronic multisystemic disease characterized by non-necrotizing granulomatous inflammation that can virtually affect any organ, although the lung is the most involved. It is a rare disease, with a variable incidence from 1 to 71 cases per 100,000 individuals per year. Its etiology is unknown, although it has been hypothesized that genetic, environmental, and lifestyle risk factors could act as trigger(s) for inflammation in genetically predisposed individuals, leading to an exaggerated, ineffective T-cellular response to putative antigens [1]; a certain role of humoral immunity in the pathogenesis of the disease has also been theorized [2]. Clinical presentation is widely heterogeneous, also depending on specific organ involvement: cough, chest discomfort, dyspnea, low-grade fever, tiredness, weight reduction, and night sweats are the most common symptoms [3]. Diagnosis is reached with histological evidence of non-necrotizing granulomas of the involved organs, in addition to typical radiological and clinical findings, and upon exclusion of other granulomatous diseases [1]. The milestone of pharmacological treatment is represented by oral corticosteroids, while the second-line therapy includes azathioprine, methotrexate, mycophenolate mofetil, cyclosporine, cyclophosphamide, leflunomide, and hydroxychloroquine; finally, a third-line therapy comprises anti-tumor-necrosis factor-α (TNF-α) antibodies, such as infliximab or adalimumab [3].

Sarcoidosis leads to an increased risk of atherosclerosis and cardiovascular disease [4, 5]. This is due to the role that inflammatory pathways play in determining endothelial dysfunction, early atherogenesis, and atherosclerotic plaque complications such as thrombosis [6].

However, data about cardiovascular risk (CV) stratification among patients with sarcoidosis based on phenotypical features are lacking.

The aim of this study is to propose a prognostic stratification model of patients with sarcoidosis through a CV risk assessment based on CV score risk and hemodynamic features of common carotid ultrasound evaluation.

## Methods

### Study population

Between March 2021 and September 2021, 53 sarcoidosis patients monitored at our regional Sarcoidosis Referral Centre were consecutively enrolled. The diagnosis of sarcoidosis was made according to international ATS/ERS/WASOG criteria [7].

A control group consisting of 48 healthy volunteers without history of respiratory or any other chronic disease and not receiving any therapy was also enrolled.

### Cardiovascular risk assessment

For each enrolled patient, CV risk was assessed using the main CV risk scores: Framingham score (FS), Heart score (HS), and American Heart Association/American College of Cardiology (AHA/ACC) score. Scores were calculated using the most recent anamnestic, laboratory, and physical findings available at the time of enrollment. Moreover, the presence of metabolic syndrome was evaluated referring to the International Diabetes Federation diagnostic criteria [8].

### Common carotid ultrasound

Each patient underwent to a complete supra-aortic trunks’ Doppler ultrasound: common carotid artery, external and internal carotid artery, and vertebral and subclavian artery were bilaterally investigated. At the level of the common carotid artery, the following hemodynamic parameters were sampled: peak-systolic velocity (PSV), end-diastolic velocity (EDV), mean velocity (Vm), pulsatility index (PI), and resistive index (RI); intima media thickness (IMT) was also sampled according to the international standardized protocols [9]. To reduce inter-operator variability, all the examinations were made by the same qualified physician, using Toshiba Aplio MX and linear probe PLT-1204AT (7–14 MHz frequency).

### Sarcoidosis patients’ stratification

Sarcoidosis patients, on the basis of the latest radiological investigations, were divided into four groups depending on the number of involved organs: 1P (“pulmonary”), with only pulmonary involvement; 1NP (“Non-pulmonary”), with a single organ involvement except lungs; 2, with two organs involved; 3, with at least three organs involved. Furthermore, clinical data about treatment regimen and the illness duration, meaning the time elapsed between diagnosis and the enrollment data, were collected. Finally, radiological classification was formulated at the time of enrollment according to Scadding criteria [10].

### Statistical analysis

Data were expressed as mean ± standard deviation (M ± SD). The Kolmogorov–Smirnov test was used for the study of sample normality. Comparisons between groups were performed by *t* test. The Spearman’s Rho test was used for correlation analysis. Contingency tables were analyzed by Fisher’s exact test. A p value less than 0.05 was considered statistically significant. Statistical analysis was obtained using SPSS Statistics 26 software.

## Results

### General features of the sample

Radiological and phenotypical features of sarcoidosis patients and demographic data of patients with sarcoidosis (*n* = 53) and controls (*n* = 48) are reported in Table 1. No significant differences in sex, age, and smoking status were found between sarcoidosis patients and controls. Almost half of sarcoidosis patients was on therapy and most of them showed a second radiological stage at the time of enrollment.

**Table 1:** Demographic features and smoking status in the sarcoidosis cohort and healthy controls; radiological and phenotypical features in sarcoidosis patients

	Sarcoidosis population	Controls	*P* value
N	53	48	
Male (%)	13 (24.5)	20 (41.6)	0.089^a^
Smoking habit			
Current (%)	2 (3.7)	7(14.5)	0.081^a^
Never (%)	51 (96.2)	41(85.4)	0.081^a^
Former (%)	0 (0)	0(0)	0.081^a^
Age (years)	56.32 ± 9.80	54.04 ± 8.27	0.106^b^
Illness duration (months)	69.15±66.57		
Treated (%)	25 (47.1) of which:		
	17 (68%): glucocorticoids alone		
	5 (20%): glucocorticoids in combination regimens		
	2 (8%): azathioprine		
	1 (4%): hydroxychloroquine		
Phenotypical classification			
1P (%)	26 (49)		
1NP (%)	3 (5.6)		
2 (%)	13 (24.5)		
3 (%)	11 (20.7)		
Radiological assessment (Scadding)			
Stage 0 (%)	3 (5.6)		
Stage 1 (%)	10 (18.8)		
Stage 2 (%)	35 (66)		
Stage 3 (%)	5 (9.4)		
Stage 4 (%)	0		

^a^Fisher’s exact test; ^b^*t* test

### Case–control comparison

Table 2 shows the main differences between sarcoidosis group and healthy controls’ group in reference to cardiovascular risk scores and Doppler, clinical, and biochemical parameters. Regarding AHA/ACC score, it was not possible to calculate it for 11 patients (8 sarcoidosis patients and 3 healthy controls), because of age, total, and HDL-cholesterol values out of the range allowed by the score itself.

**Table 2:** Case–control comparison: cardiovascular risk scores and Doppler, clinical, and biochemical parameters

	Sarcoidosis patients	Controls	*P* value
Cardiovascular risk scores			
Framingham score	7.99 ± 4.85	5.59 ± 5.05	0.008^a^
AHA/ACC score	8.33 ± 7.72	3.40 ± 4.19	0.000^a^
Heart score	1.50 ± 0.84	1.20 ± 0.79	0.034^a^
Doppler parameters			
Pulsatility index	1.51 ± 0.37	1.49 ± 0.36	0.285^a^
Resistivity index	0.70 ± 0.06	0.69 ± 0.07	0.101^a^
Peak systolic velocity (cm/sec)	74.35 ± 17.95	80.74 ± 19.78	0.045^a^
End diastolic velocity (cm/sec)	19.78 ± 5.21	22.25 ± 6.39	0.017^a^
Mean velocity (cm/sec)	25.72 ± 6.26	27.95 ± 7.05	0.047^a^
Intima media thickness (mm)	0.76 ± 0.19	0.70 ± 0.10	0.016^a^
Clinical and biochemical parameters			
Total cholesterol (mg/dl)	200.03 ± 46,82	187.52 ± 33,97	0.065^a^
LDL-cholesterol (mg/dl)	120.15 ± 38.37	109.22 ± 30.10	0.058^a^
HDL-cholesterol (mg/dl)	57.66 ± 17.69	57.97 ± 18.18	0.464^a^
Triglycerides (mg/dl)	132.60 ± 51.08	104.29 ± 64.03	0.007^a^
Glucose (mg/dl)	98.28 ± 22.11	86.43 ± 10.18	0.000^a^
Systolic blood pressure (mmHg)	128.11 ± 16.38	119.27 ± 12.96	0.001^a^
Diastolic blood pressure (mmHg)	78.49 ± 8.52	76.35 ± 11.51	0.144^a^
Waist circumference (cm)	99.43 ± 12.95	95.48 ± 14.89	0.078^a^
Metabolic syndrome (n°)	31	7	0.000^b^

^*a*^*t *test

^b^Fisher’s exact test

In the context of cardiovascular risk scores, each one (Framingham score, AHA/ACC score, and Heart score) was significantly higher in sarcoidosis than in healthy controls (*p* = 0.008, *p* = 0.000, *p* = 0.034 respectively). Regarding Doppler parameters, PSV, EDV, and Vm were significantly lower in sarcoidosis cohort (*p* = 0.045, *p* = 0.017, *p* = 0.047 respectively), whereas IMT showed higher values in sarcoidosis group than in controls (*p* = 0,016); no differences were found when considering PI and RI values (*p* = 0.285 and *p* = 0.101, respectively). Finally, in the context of clinical and laboratoristic parameters, triglycerides, glucose, and systolic blood pressure values were higher in sarcoidosis group than in controls (*p* = 0.007, *p* = 0.000, and *p* = 0.001, respectively).

Finally, the prevalence of metabolic syndrome was significantly higher in sarcoidosis subjects than in healthy controls (31 versus 7, *p* = 0.000). In detail, of the 31 sarcoidosis patients who suffered from metabolic syndrome, at the time of the enrollment 14 were receiving pharmacological therapy for sarcoidosis: 12 of them were taking glucocorticoids (alone or in combination regimens), 1 was taking azathioprine, and 1 hydroxychloroquine. Of the 17 remaining sarcoidosis patients who did not receive pharmacological therapy at the time of enrollment, 15 of them previously received at least a cycle of glucocorticoids-based therapy during disease history.

### Analysis of sarcoidosis clinical phenotypes

Evaluating the clinical phenotypes of sarcoidosis patients (excluding the phenotype 1NP for too small sample size), no differences were found in relation to cardiovascular risk scores when phenotypes were considered per couple (Table 3). Conversely, the analysis of Doppler parameters shows: PI and RI values were significantly higher in 2-phenotype than in 1P-phenotype (*p* = 0.003 and *p* = 0.027 respectively); an EDV value was significantly lower in 2-phenotype than in 1P-phenotype (*p* = 0.048) and 3-phenotype (*p* = 0.040); an IMT value was significantly higher in 2-phenotype than in 3-phenotype (*p* = 0.007).

**Table 3:** Analysis of cardiovascular risk scores and Doppler parameters in sarcoidosis clinical phenotypes

	Framingham score	AHA/ACC score	Heart score	Pulsatility index	Resistivity index	Peak systolic velocity (cm/s)	End diastolic velocity (cm/s)	Mean velocity (cm/s)	Intima media thickness (mm)
Clinical phenotype									
1P	8.33±4.70	8.70±7.51	1.53±0.90	1.39±0.28	0.69±0.05	69.76±13.92	20.4±4.72	25.48±5.41	0.75±0.18
2	7.83±5.99	8.63±10.40	1.46±0.77	1.76±0.53	0.73±0.08	78.22±24.44	17.61±4.96	23.85±6.78	0.81±0.17
3	8.03±4.61	5.83±5.40	1.45±0.82	1.49±0,24	0.71±0.04	81.42±17.63	21.81±6.29	28.80±7.43	0.66±0.08
Phenotypes comparison (*p* value of *t* test)									
1P versus 2	0.387	0.491	0.397	0.003	0.027	0.087	0.048	0.209	0.087
1P versus 3	0.430	0.134	0.396	0.163	0.163	0.396	0.228	0.068	0.065
2 versus 3	0.463	0.223	0.491	0.066	0.180	0.087	0.040	0.051	0.007

Furthermore, the analysis of the influence of biochemical or clinical parameters on cardiovascular risk when evaluated by Framingham score (Table 4) highlighted a series of phenotype-specific correlations: a direct correlation with glucose in 1P-phenotype (*R* = 0.469; *p* = 0.015); an inverse correlation with total and HDL-cholesterol (*R* = − 0.615, *p* = 0.025 and *R* = − 0,605, *p* = 0.028 respectively) and a direct correlation with glucose (*R* = 0.661, *p* = 0.013) in 2-phenotype; a direct correlation with systolic blood pressure in 3-phenotype (*R* = 0.874, *p* = 0.000).

**Table 4:** Correlation between biochemical and clinical parameters and Framingham score in sarcoidosis clinical phenotypes (Rho’s Spearman test)

	1P-phenotype		2-phenotype		3-phenotype	
	R	*P* value	*R*	*P* value	*R*	*P* value
Total cholesterol	− 0.003	0.9847	− 0.6154	0.0251	− 0.0963	0.7781
LDL-cholesterol	0.1224	0.5511	− 0.3124	0.2986	− 0.0776	0.8205
HDL-cholesterol	− 0.2524	0.2134	− 0.6053	0.0283	− 0.3607	0.2757
Triglycerides	0.1448	0.4803	− 0,3654	0.2194	0.1968	0.5619
Glucose	0.4693	0.0155	0.6611	0.0138	0.4449	0.1702
Systolic blood pressure	0.3523	0.0775	0.5174	0.0701	0.8745	0.0004
Diastolic blood pressure	0.0946	0.6454	0.1917	0.5302	0.4199	0.1984
Waist circumference	0.1073	0.6015	0.3152	0.2941	0.5251	0.0971

### Influence of gender, therapy, and illness duration

With reference to gender (Table 5), only Heart Score detected a statistically significant difference between males and females with higher values in the first group (*p* = 0.012); about therapy, only Framingham score underscored a lower cardiovascular risk in treated group than in non-treated one (*p* = 0.038), while no differences were found in the case of AHA/ACC score, Heart Score, or analyzing Doppler parameters. Illness duration did not correlate with cardiovascular risk scores, while an inverse correlation was found between illness duration and two Doppler parameters: PSV (*R* = − 0.298; *p* = 0.030) and EDV (*R* = − 0.396; *p* = 0.003).

**Table 5:** Influence of gender, therapy, and illness duration on cardiovascular risk

	Gender			Therapy			Illness duration	Framingham score
	Male	Female	*P* value_a_	Treated	Untreated	P value_a_	(R; *p* value_b_)	(R; *p* value_b_)
Cardiovascular risk scores								
Framingham score	8.62±4.38	7.22±5.03	0.220	6.58±4.58	9.11±4.79	0.038	0,160; 0,252	
AHA/ACC score	9.64±8.37	7.61±7.15	0.062	7.21±6.29	10.12±8.85	0.091	0.016; 0.912	
Heart score	1.76±0,96	1.25±0.62	0.012	1.37±0.79	1.60±0.87	0.189	0.039; 0.776	
Doppler parameters								
Pulsatility index				1.47±0.36	1.55±0.38	0.231	− 0.089; 0.523	0.004; 0.975
Resistivity index				0.70±0.06	0.72±0.06	0.112	− 0.077; 0.583	0.005; 0.968
Peak systolic velocity				76.15±19.86	72.75±16.26	0.248	− 0.298; 0.030	− 0.268; 0.051
End diastolic velocity				20.80±4.55	18.87±5.65	0.089	− 0.396; 0.003	− 0,275; 0.004
Mean velocity				26.73±6.11	24.83±6.36	0.137	− 0.221; 0.111	− 0.406; 0.002
Intima media thickness				0.73±0.18	0.78±0.19	0.162	− 0.089; 0.523	0.429; 0.0011

^a^*t* test;

^b^Rho’s Spearman test

On the other hand, Doppler parameters correlated themselves with Framingham score: in detail, EDV and Vm showed an inverse correlation with Framingham score (*R* = − 0.275, *p* = 0.004; *R* = − 0.406, *p* = 0.002, respectively), whereas IMT showed a direct one (*R* = 0.429; *p* = 0.001).

## Discussion

Sarcoidosis is a chronic granulomatous disease that can lead to an increased risk of cardiovascular diseases through several mechanisms.

In the first place, a key role is probably played by an inflammation-associated premature atherosclerosis: at this regard, Libby [6] underlined the important influence of inflammation in the development of vascular atheroma: pro-inflammatory cytokines, such as interleukin 1β (IL-1β) or TNF- α, induce the expression of vascular cell adhesion molecule-1 (VCAM-1) which promotes an early adhesion of mononuclear leukocytes (namely monocytes and T-lymphocytes) to arterial endothelium at sites of atheroma initiation. In the same way, transcriptional activation of VCAM-1 gene is partially mediated by transcription nuclear factor-κB (NF- κB), notoriously recognized as a pivotal mediator of inflammatory responses [11]. Moreover, interleukin 8 (IL-8) may act as a leukocyte chemoattractant during atherogenesis, while interferon-γ (IFN-γ) could stimulate the release of chemokines involved in lymphocyte recruitment. Finally, the author argued that inflammation could also have a role in the progression of the atheroma and in determining its complications: for example, inflammatory mediators can promote atherosclerotic plaque instability through the expression and the activation of matrix metalloproteinases (MMPs) and limiting collagen production by smooth muscle cells.

On the other hand, chronic inflammation is capable to promote initiation and propagation of coagulation cascade, downregulate anti-coagulant pathway, and inhibit fibrinolysis, leading to an increased risk of vascular thrombosis [5].

Moreover, a genetic predisposition shared between immune-mediated inflammatory diseases and CV diseases could be hypothesized. For example, HLA-DRB1 seems to predispose to development of rheumatoid arthritis inducing the selection of autoreactive CD4 + T cells which can be intriguingly found in both synovial tissue and atherosclerotic plaque. Similarly, it has been found that HLA-DRB1*1101 could be considered as a risk factor for development of sarcoidosis, again suggesting the hypothetical connection role of genetics [5].

Finally, an increased risk of CV disease among sarcoidosis patients could arise from medical treatments: glucocorticoids, which represent the milestone of therapy in sarcoidosis management, can often contribute to the development of CV disease risk factors, such as diabetes mellitus, arterial hypertension, and dyslipidemia, especially in the case of their long-term use [12].

As proof of the strong relationship between sarcoidosis and CV risk, a recent retrospective cross-sectional study on a large Israeli cohort of 3750 sarcoidosis patients and 18,139 age- and sex-matched controls highlighted that sarcoidosis was associated to ischemic heart disease (adjusted odds ratio 1.5; 95% confidence interval 1.36–1.66) and to increased risk for all-cause mortality compared to controls (adjusted hazard ratio 1.93; 95% confidence interval 1.76–2.13) [13].

In light of these considerations, the aim of this observational study has been to analyze cardiovascular risk in a cohort of sarcoidosis patients, evaluating the possibility of creating a prognostic stratification model based on clinical phenotyping that could help physicians to timely identify patients at high risk of CV diseases development.

As expected from scientific literature [5], our data showed that cardiovascular risk in the sarcoidosis group was higher than in the healthy controls group when evaluated by cardiovascular risk scores. On the other hand, also the analysis of subclinical atherosclerosis performed through carotid Doppler ultrasound examination showed a higher CV risk in the sarcoidosis group. In particular, PSV and EDV were significantly lower in sarcoidosis cohort, while IMT values showed an opposite trend, suggesting anyway a higher CV risk in this group. At this regard, several studies demonstrated that reduction of PSV and EDV or IMT increase are related to higher CV risk [14–16]. Mean velocity seems to follow the same trend of PSV and EDV, presenting lower values in the sarcoidosis group: even if an accurate role of common carotid artery mean velocity has not yet been described, mean velocity reduction could represent a risk factor for cerebrovascular events, as we can consider it as an expression of impaired cerebrovascular arterial flow.

Interestingly, Doppler parameters showed a correlation with Framingham score and illness duration. In the first case, an inverse correlation for EDV and mean velocity and a direct correlation for IMT toward Framingham score was identified: these correlations show that the same Doppler parameters’ variations suggestive of increased CV risk could also intercept, with the same meaning, a higher Framingham score. This finding confirms the enormous usefulness of carotid Doppler ultrasound in easily performing a CV risk assessment of sarcoidosis patients, so intercepting who could benefit from a closer follow-up and a tailored pharmacological therapy oriented to the control of the disease and cardiovascular risk factors. In the second case, an inverse correlation was found between PSV and EDV and illness duration, so suggesting an increased risk in the patients in which sarcoidosis has been diagnosed for a longer time: this could be intuitively explained referring to the role that chronic inflammation plays in determining a continue and progressive atherosclerotic damage, exposing the patient with an old history of the disease at a higher CV risk.

A higher CV risk of sarcoidosis patients has emerged even when evaluating the prevalence of metabolic syndrome that represents itself an important risk factor for cardiovascular disease [17]: it was significantly higher in sarcoidosis subjects than in healthy controls and triglycerides, glucose and systolic blood pressure values were predictably higher in the same group. Higher prevalence of metabolic syndrome among sarcoidosis patients can be due mainly to two factors: first, metabolic syndrome is a known risk factor for developing sarcoidosis [18]; furthermore, sarcoidosis patients experience side effects of systemic corticosteroids, such as arterial hypertension and diabetes. Analyzing our sarcoidosis sample, almost half of sarcoidosis patients who suffered from metabolic syndrome were taking therapy for the disease and almost all of patients who were not taking therapy at the time of enrollment underwent previously a cycle of glucocorticoid-based pharmacological therapy. Even if the influence of drug therapy on the development of metabolic syndrome can be reasonably considered, sarcoidosis should be probably considered an independent risk factor for metabolic syndrome, regardless of the treatment regimen [19].

In the setting of sarcoidosis clinical phenotypes, the analysis of CV risk scores did not show any statistically significant difference among them; however, a possible partial difference in CV risk was suggested by hemodynamic and morphological parameters of carotid Doppler ultrasound. In detail, 2-phenotype presented a higher risk than 1P-phenotype for the presence of significantly higher PI and RI values and a significantly lower EDV value [20, 21]; furthermore, 2-phenotype presented a higher risk than 3 as suggested by the presence of significantly higher IMT value.

Another interesting observation emerging from clinical phenotyping is that each sarcoidosis phenotype shows a specific correlation between a biochemical or clinical parameter and the Framingham score: glucose in 1P-phenotype, total and HDL-cholesterol, and glucose in 2-phenotype and systolic blood pressure in 3-phenotype.

Even if the reason of these differences between phenotypes seems to be unclear, these data suggest an important consideration: CV risk does not appear to be linked to the number of organs involved by the disease, but rather to peculiar phenotypical features of the patients that is necessary to identify and better characterize, to plan a customized therapy and follow-up strategy responding to the exact clinical needs of the patient.

Finally, an important consideration must be done about the influence of therapy on CV risk of sarcoidosis patients. At this regard, in our cohort, no substantial differences in terms of CV risk between treated and not-treated groups were found; only Framingham scores values resulted significantly lower in the treated group than in not-treated one, but nor the other CV risk scores neither Doppler parameters’ evaluation supported this data. These considerations seem to suggest overall that chronic inflammation in sarcoidosis behaves as a steady, active cardiovascular risk factor, regardless of therapeutic regimen taken by the patient.

Concluding, we propose a prognostic stratification model of sarcoidosis patients in which CV risk assessment could be performed through an integration of CV risk scores with some specific common carotid Doppler ultrasound parameters, namely EDV, PSV, IMT, and mean velocity. In fact, our results showed that Doppler parameters can be capable to underline differences in terms of subclinical atherosclerosis with greater accuracy than CV risk scores. Moreover, these mentioned Doppler parameters seem to hold an important prognostic value due to their relationship with Framingham score and illness duration. Anyway, further studies are necessary to define specific cut-off values of Doppler parameters relative to different CV risk ranges.

Our study represents the first attempt to investigate how CV risk is distributed in a cohort of sarcoidosis patients subdivided on the basis of clinical features. Results suggest an innovative possibility of using hemodynamic Doppler parameters to early identify a higher CV risk at a subclinical level; this is also considering that our data demonstrated for the first time a linkage between EDV, mean velocity, IMT, and Framingham score risk. The analysis of Doppler parameters themselves, in addition to biochemical and clinical findings, proved to be useful to identify some differences of CV risk among sarcoidosis clinical phenotypes: seemingly, CV risk could depend on specific clinical features of the patients. However, data need to be expanded and confirmed.

The main limit of our study is represented by small sample size that may have led to partial results. This may have been caused also by splitting the cohort of patients with sarcoidosis in several phenotypes. For these reasons, it is necessary to expand sample size to confirm and better define our data. Results could help clinicians to improve the management of sarcoidosis patient by enlarging therapeutic focus also toward the correct and early monitoring of their cardiovascular risk.

## Data Availability

All data supporting the findings of this study are available from corresponding author on reasonable request.
